# Interpretation of chest fluoroscopy: the risk of misdiagnosing atelectasis as pneumothorax due to greyscale inversion

**DOI:** 10.1002/anr3.12307

**Published:** 2024-06-01

**Authors:** C. Y. Lin, N. B. Cavanaugh, S. Subramani, A. K. Singhal, S. Hanada

**Affiliations:** ^1^ Department of Anesthesiology and Perioperative Care School of Medicine, University of California, Irvine School of Medicine Irvine CA USA; ^2^ Department of Anesthesia University of Iowa Iowa City IA USA; ^3^ Department of Cardiothoracic Surgery University of Iowa Iowa City IA USA

**Keywords:** atelectasis, fluoroscopy, pneumothorax, radiograph, X‐rays

## Abstract

Anaesthetists may be required to work in hybrid theatres for procedures using fluoroscopic imaging. Adequate knowledge of fluoroscopic images allows prompt and effective emergency management of complications which arise during procedures. Here, we present a case of severe hypotension and hypoxia occurring shortly after induction of anaesthesia. Atelectasis was mistaken for a pneumothorax due to misinterpretation of fluoroscopic imaging, which demonstrated a dark pleural cavity peripheral to a partially collapsed left lung, leading to an incorrect diagnosis. This case highlights the importance of understanding greyscale inversion in fluoroscopy.

## Introduction

Fluoroscopic imaging is commonly used either with a ‘C‐arm’ or in designated satellite suites or hybrid theatres with embedded fluoroscopy imaging machines [1, 2]. Fluoroscopy is a convenient tool for capturing images when other means, such as X‐ray radiography and computed tomography are not readily accessible. Because of this, fluoroscopy may be especially useful in the prompt diagnosis and management of emergencies [3, 4]. Here, we present a patient who developed severe hypotension shortly after induction of anaesthesia accompanied by peripheral oxygen desaturation and high airway pressures refractory to conventional treatment. While fluoroscopic imaging was readily available, the images were misinterpreted as a pneumothorax rather than atelectasis. This demonstrates the importance of understanding images obtained using fluoroscopy versus more familiar imaging modalities. Written informed consent was obtained from the patient for the publication of this case report.

## Case report

A 52‐year‐old man (height 195 cm, weight 112 kg) presented for elective transcatheter aortic valve replacement (TAVR) and mitral valve replacement (TMVR). The patient had severe prosthetic mitral valve stenosis and severe paravalvular prosthetic aortic valve regurgitation with moderate prosthetic aortic stenosis. His cardiac history consisted of surgical aortic and mitral valve replacements for infective endocarditis four years previously, hypertension, hyperlipidaemia, paroxysmal atrial fibrillation with a history of left atrial appendage thrombus, chronic diastolic heart failure and left ventricular outflow tract pseudoaneurysm. He had recurrent infective endocarditis three years after his initial episode which was treated with antibiotics. He had stable cardiac function until recent worsening, for which he underwent this procedure.

**Figure 1:**
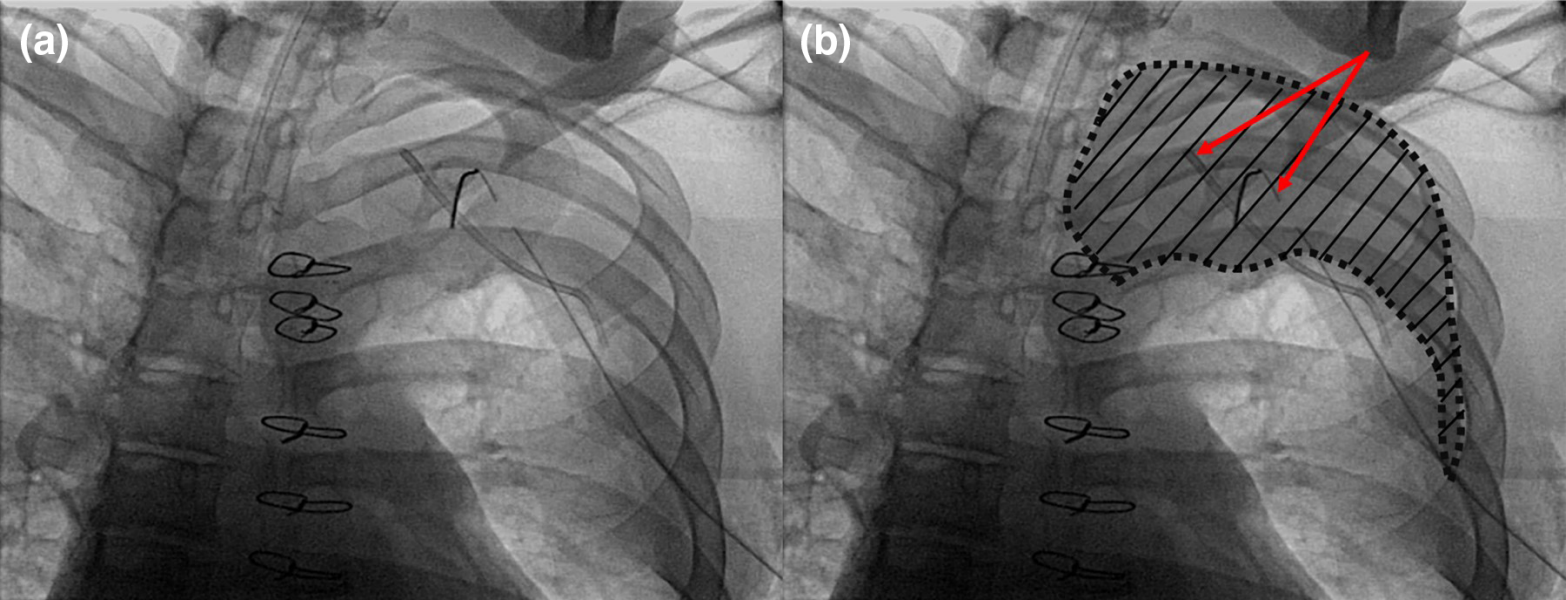
Identical fluoroscopic images of the chest taken after the placement of a pigtail catheter and chest tube. (a) Unlabelled image. (b) On fluoroscopic images, the darker radiopaque area in the apical field peripheral to the partially collapsed lung in the left upper lobe (grey shaded area with black lines), indicates the absence of air, suggesting atelectasis rather than pneumothorax. Red arrows indicate the pigtail catheter and chest tube.

The patient was positioned supine on the procedure table in the cardiac catheterisation laboratory suite. Invasive arterial monitoring was established with a radial artery catheter before induction of general anaesthesia. His vital signs were stable: blood pressure 160/76 mmHg, heart rate 87 beats.min^−1^ and a respiratory rate of 20 breaths.min^−1^. He was pre‐oxygenated to 100% SpO_2_ and general anaesthesia was induced with 100 mcg of fentanyl and 150 mg of propofol. Sixty milligrams of rocuronium was given and his trachea was intubated without difficulty. Immediately following tracheal intubation, the patient became increasingly hypotensive (to around 70/35 mmHg) and hypoxic (to 80% SpO_2_) with increased airway pressures (to 45 cmH_2_O). In response, a norepinephrine infusion was initiated, at a rate up to 0.2 μg.kg^−1^.min^−1^, and a 50 μg bolus of epinephrine was also given intravenously. Lung sounds were diminished bilaterally, especially in the left lung, and mild wheezing was heard. Salbutamol was administered via the tracheal tube from a metered dose canister due to suspicion of bronchospasm. Despite efforts to stabilise the patient's circulatory and respiratory systems, the oxygen saturation continued to decrease to 70% and blood pressure also decreased to approximately 60/30 mmHg. As the angiographic imaging system (Artis Zee, Siemens Healthcare, Germany) was readily available in the cardiac catheterization suite, fluoroscopic images of the chest (Fig. 1) were quickly obtained to investigate the possibility of endobronchial intubation and pneumothorax. A partially collapsed left upper lobe surrounded by a darker radiopaque field was seen, which was interpreted by the attending anaesthetist and attending interventional cardiologist as air in the apical pleural space.

The cardiothoracic surgical team was immediately consulted, and an attending cardiac surgeon initially attempted a needle decompression to treat the presumed pneumothorax, but this led to no improvement. The cardiothoracic surgeon then placed an 8 French pigtail catheter (Merit Medical, Utah, USA), followed by a chest tube (Atrium Medical Corporation, New Hampshire, USA) into the fourth intercostal space on the left. Again, there was no evacuation of air. Due to this unexpected finding, the fluoroscopic images were reviewed again by the same attending anaesthetist and interventional cardiologist, along with the cardiac surgeon, who then diagnosed atelectasis rather than pneumothorax (Fig. 1). Having diagnosed atelectasis, gentle recruitment manoeuvres were applied with careful monitoring, leading to gradual improvements in haemodynamics. The patient continued to be intermittently hypotensive, with systolic blood pressure ranging from 70 to 100 mmHg during these events in the procedure room and remained on the norepinephrine infusion at 0.05–0.1 μg.kg.min^−1^. Although his haemodynamic status and oxygen saturation were stabilised approximately 30 min after induction of anaesthesia, following the correct diagnosis of atelectasis, the planned procedure was abandoned. The patient was transferred to the cardiovascular intensive care unit for continued monitoring and care, sedated with midazolam and fentanyl, and ventilated at a tidal volume of 450 ml, a respiratory rate of 16 breaths.min^−1^ and a positive end‐expiratory pressure (PEEP) of 5 cmH_2_O. The patient's trachea was extubated and vasopressor support was weaned off two days later. He was then transferred to ward‐based care on the cardiology inpatient floor. He underwent successful TAVR seven days later, and TMVR one month later.

## Discussion

This report describes a case where atelectasis was misdiagnosed with a pneumothorax shortly after anaesthetic induction due to misinterpretation of fluoroscopic images taken in an emergency. Fluoroscopic imaging showed a darker radiopaque apical field peripheral to a partially collapsed left upper lobe of the lung. On standard radiographs, which anaesthetists have more experience with, areas that appear dark indicate less dense material, such as air [5, 6]. Therefore, because of the dark periphery of the left upper thorax in the images (Fig. 1) captured during the procedure outlined in this case report, clinicians unaccustomed to interpreting fluoroscopic images could easily mistake this for a pneumothorax. It is important to note that fluoroscopic images, such as those produced with an angiographic system or mobile C‐arm, are digitally manipulated to appear with an inverted greyscale compared to that of standard radiographs [7]. Thus, the dark or radiopaque field peripheral to the collapsed left upper lobe in this acquired fluoroscopic image indicated the absence of air in the thorax. This indicates the importance of understanding that air is white in fluoroscopy, whereas it is dark grey in standard radiographs [5, 6]. Both the interventional cardiologist and cardiac surgeon involved had extensive experience with fluoroscopic imaging due to their frequent use of it in procedures. In contrast, the anaesthetist involved was less familiar with interpreting fluoroscopic imaging although they frequently work in this setting. This underscores the need for training in fluoroscopic interpretation across all relevant specialties, not just those who regularly use this technology. Such training would ensure accurate diagnoses and prevent misinterpretation, potentially improving patient outcomes.

In this case, refractory severe hypotension, hypoxia and high airway pressures occurred immediately after induction of anaesthesia. There were a number of possible causes, including overdosing of anaesthetic agents, bronchial intubation, anaphylaxis, bronchospasm and pulmonary embolism, or a combination of these, as well as atelectasis and pneumothorax. While tension pneumothorax is an unlikely aetiology in this scenario due to the absence of predisposing factors such as bullous emphysema, it was still essential to rule out pneumothorax in this unanticipated haemodynamic disturbance. In this particular case, the haemodynamic disturbance was likely caused by significant valvular disease with chronic heart failure, compounded by the effect of general anaesthesia. Pre‐existing bilateral effusions with atelectasis, revealed in the postoperative evaluation, also likely contributed to events.

It is crucial for anaesthetists to be able to diagnosis and troubleshoot problems quickly and efficiently in cases where complications arise under anaesthesia. There are many devices and monitors which help with the differential diagnosis should a patient become unstable. However, diagnostic fluoroscopy may be one area in which anaesthetists do not have extensive training or experience [8]. Fluoroscopy can be a useful emergency imaging tool because it is often readily available in or near theatres and embedded within hybrid operating rooms and procedure suites, such as in the cardiac catheterisation laboratory. It is therefore important that anaesthetists have adequate knowledge of this imaging modality. Although point‐of‐care ultrasound is another useful and reliable tool to rapidly diagnose a pneumothorax [9, 10], in our case, capturing a fluoroscopic image by the interventional cardiologist was a quicker investigation, while the anaesthetists were stabilising the acutely unwell patient.

To our knowledge, there are no previous reports describing similar incidents involving misinterpretation of fluoroscopic images causing misdiagnosis of pneumothorax. Furthermore, an extensive literature search found little discussion about the greyscale inversion in fluoroscopy. It is crucial for anaesthetists to be aware of this in order to make correct diagnoses and institute appropriate emergency management, and to avoid unnecessary invasive procedures. The multidisciplinary nature of the team involved in this case was crucial. Team members with extensive fluoroscopy experience shared their knowledge, which was invaluable in reaching the correct diagnosis. This underscores the importance of a diverse and experienced team for accurate diagnoses and effective patient care. To prevent similar future incidents, regular training on fluoroscopic interpretation, such as greyscale inversion, is essential. At our institution, periodic didactic lectures have been implemented to keep relevant staff informed about common issues and best practices in fluoroscopic interpretation. Additionally, targeted educational initiatives can facilitate early and accurate management of critical conditions, fostering a culture of continuous learning and collaboration.
